# Prevalence of smoking during pregnancy and associated risk factors among Canadian women: a national survey

**DOI:** 10.1186/1471-2393-10-24

**Published:** 2010-05-24

**Authors:** Ban Al-Sahab, Masarat Saqib, Gabriel Hauser, Hala Tamim

**Affiliations:** 1 School of Kinesiology and Health Science, York University, Toronto, ON Canada M3J 1P3

## Abstract

**Background:**

Cigarette smoking carries a threat both to the expecting mother and her newborn. Data on the prevalence and predictors of smoking during pregnancy is limited in Canada. Canadian studies are mainly representative of specific cities and/or provinces. Therefore, the study aims to assess the prevalence of smoking during pregnancy and its associated risk factors throughout the Canadian provinces and territories.

**Methods:**

The analysis was based on the Maternity Experience Survey targeting women aged ≥15 years who had singleton live births during 2005/06 in the Canadian provinces and territories. The outcome was ever smoking during the thirst trimester of pregnancy. Socio-economic factors, demographic factors, maternal characteristics, and pregnancy related factors that proved to be significant at the bivariate level were considered for a logistic regression analysis. Bootstrapping was performed to account for the complex sampling design.

**Results:**

The sample size was 6,421 weighted to represent 76,508 Canadian women. The prevalence of smoking during pregnancy was 10.5%, whereby smoking mothers consumed on average 7 cigarettes a day (95% Confidence interval - CI: 6.5-7.4; SD = 5.7). Regression analysis revealed that mothers who smoked during pregnancy were more likely to be of low socio-economic status, non-immigrant, single and passive smokers during pregnancy. Not attending prenatal classes and experiencing stressful events before/during pregnancy also increased the mothers' odds of smoking during pregnancy. While the age of the mother's first pregnancy was negatively associated with smoking during pregnancy, the mother's current age was positively associated with it.

**Conclusion:**

Smoking during pregnancy is still prevalent among Canadian women. The findings may be useful to enhance smoking prevention programs and integrated health promotion strategies to promote positive health behaviors among disadvantaged pregnancies.

## Background

Cigarette smoking carries a threat both to the expecting mother and her newborn. Tobacco products are responsible for many complications including tobacco-induced abortions, deaths from perinatal disorders [1], newborns requiring admission to neonatal intensive care unit [2], low-birth weight infants [3], and deaths from Sudden Infant Death Syndrome [4]. Furthermore, cigarette smoking increases the risk of infertility and conception delay [5], as well as harmful pregnancy outcomes, such as premature rupture of membranes [6], placenta previa [7], abruptio placenta [8], stillbirth [4], and preterm delivery [9].

The prevalence of smoking among pregnant women has been shown to vary across different countries. For example, prevalence rates range from 9.9% in Japan [10], to 17% in Australia [11], to 30-35% in Spain [12]. In Canada, the prevalence of smoking among pregnant women was estimated to be 17% in 2000/01 [13]. During the same period, however, a study conducted in three Southern Ontario Health Units revealed that 10.4% of the women smoked at some point during their pregnancy [14]. Data taken from 1999-2000 revealed that, in Winnipeg, 26.2% of non-aboriginal and 61.2% of aboriginal women smoked after being aware of their pregnancy [15]. From 1998-2001, the overall prevalence of smoking just before delivery in Nova Scotia was 25.1% [16].

Internationally, predictors of smoking among pregnant women have been well investigated. Studies found that age, education, ethnicity, martial status, alcohol consumption, work status, and the mother's reproductive history are associated with smoking during pregnancy [11,17-19]. In Canada, pregnant smokers were more likely to be under 25 years of age [14], to have lower income levels [20], to be unmarried [20] and to have others in the household who smoked [21]. Moreover, Canadian women who were pregnant with their first child and who consumed alcohol during their pregnancy were also more likely to relapse to smoking during pregnancy [22].

Despite the well known and detrimental effects of smoking, it remains prevalent among pregnant women. In order to determine the groups of women that are at higher risk of smoking and to tailor appropriate interventions, the identification of prevalence and predictors is crucial. Data on the prevalence and predictors of smoking during pregnancy, however, is limited in Canada. Canadian studies are mainly representative of specific cities and/or provinces [14,15,20,21,23-30]. To our knowledge, only two studies assessed smoking during pregnancy at the national level [13,22]. The study by Connor & McIntyre (1999) used 1994 data from the National Longitudinal Child and Youth Survey, whereby the unit of analysis is the child and not the mother [22]. Millar & Hill (2004), on the other hand, based the analysis on the 2000/01 Canadian Community Health Survey [13]. Smoking during pregnancy, however, was assessed within 5 years in the past, which increases the chance of recall bias. Both national studies excluded mothers in the northern territories and investigated limited demographic and socio-economic predictors. The present study, however, uses data from a recent specialized survey on pre and post delivery experiences among mothers residing in both the Canadian provinces and the territories. It aims to examine and assess the prevalence of smoking during pregnancy and the potential socio-economic, demographic maternal and pregnancy related risk factors.

## Methods

The analysis of this study is based on the Maternity Experience Survey (MES) that was sponsored by Public Health Agency of Canada and conducted by Statistics Canada in 2006. The MES study is a nationwide survey that assessed pregnancy, delivery and postnatal experiences of mothers and their children. Participants eligible for the study were women aged 15 years and above, who had singleton live births between the period of February 15, 2006 and May 15, 2006 in the provinces of Canada and between November 1, 2005 and February 1, 2006 in the territories of Canada and who lived with their baby at the time of data collection. A stratified random sample of 8,542 Canadian women was selected without replacement from the 2006 Canadian Census of Population. Around 8,244 women were estimated to have met the eligibility criteria of the study. A total of 6,421 women, however, responded to the survey. Non-response to the survey was mainly from inability to establish contact with the mothers. Prior to data collection, an introductory letter and survey pamphlet were mailed to the women and invited them to participate in the survey. Then the data was collected through telephone interviews using a computer-assisted telephone interview application. In an attempt to recruit the highest number of mothers possible, a total of 25 calls per each case were made during different days of the week and different hours of the day. The MES questionnaire was also available in 15 languages. Majority of the interviews were conducted between the 5^th ^and 9^th ^month after delivery and lasted on average 45 minutes. The MES project was presented to Health Canada's Science Advisory Board, Health Canada's Research Ethics Board and the Federal Privacy Commissioner and was approved by Statistics Canada's Policy Committee. The study has been previously described in other references [31,32].

The main outcome of the study is smoking during pregnancy defined as ever smoking during the last three months of pregnancy. This variable was measured based on the question "During the last 3 months of your pregnancy, did you smoke daily, occasionally, or not at all?" Daily and occasional respondents were grouped as ever smokers. Other considered smoking related variables were smoking before pregnancy assessed by the question "In the three months before your pregnancy, or before you realized you were pregnant, did you smoke daily, occasionally or not at all?" and smoking after pregnancy measured by the question "At the present time, do you smoke cigarettes daily, occasionally or not at all?". The response categories for both questions were daily, occasionally and not at all. Similar to smoking during pregnancy, daily and occasional respondents were grouped as smokers. The number of cigarettes smoked was also considered. For daily users, the question was: "How many cigarettes do you smoke each day?". For occasional smokers, on the other hand, it was: "On the days that you do smoke, how many cigarettes do you usually smoke?".

A wide range of independent variables were investigated as potential predictors of smoking during pregnancy. These variables were: i) socio-economic factors: maternal years of education, total household income, maternal work status during pregnancy and place of residence (urban vs. rural); ii) demographic factors: immigration status and province of residence; iii) maternal characteristics: marital status, age at first pregnancy, number of previous pregnancies, age at selected birth, and mother's perceived health; and iv) pregnancy related factors: self reported weight gain during pregnancy, alcohol drinking during pregnancy, support during pregnancy, mother's reaction to pregnancy, mother's stress level before and during pregnancy, health problems during pregnancy (defined as any new medical conditions or health problems that required taking medication for more than 2 weeks, having special care, or extra tests), passive smoking during pregnancy (defined as ever living with someone who smoked at any point during the mother's pregnancy), attendance of prenatal or childbirth education classes during the pregnancy and the number of times the pregnant women have visited a health care provider during the pregnancy. All the variables, except for mother's stress level, were directly self-reported by the mother. The mother's stress level, however, was measured through a set of 13 questions that examined the mother's experience of 13 specific stressful events in the past 12 months before the birth of her selected child. The answers for these questions were categorised as "Yes" or "No". Consequently, the sum of the "Yes" responses was calculated for each mother to represent the number of stressful events experienced [32]. The stress questions were adapted by Pregnancy Risk Assessment Monitoring System from the Life Events Inventory that was developed by Newton and Hunt [33]. For more information, the MES questionnaire is available online [34].

The prevalence of smoking was estimated through population weights and examined across all the Canadian provinces and territories. Population weights estimate the number of people not selected in the sample that have been represented by each person in the sample. It also takes into consideration non-response in the survey [32]. At the bivariate level, differences in the proportion of smokers was assessed among the different levels of each predictor using normalized weights. Odds ratios (OR) using 95% confidence intervals (95% CI) were performed for categorical variables. Differences in means with 95% confidence interval estimations, on the other hand, were employed for continuous variables. Factors that proved to be significant at the bivariate level were considered for a multivariate logistic regression analysis. Adjusted OR and 95% CI were reported for the final model. To account for the complex sampling design, bootstrapping was performed to calculate all the 95% CI estimates [35,36]. Population weights, normalized weights and bootstrap weights were all created by Statistics Canada and provided with the MES data file. All analyses, in exception to bootstrapping, were conducted using the Statistical Package for Social Sciences (SPSS, version 16.0). Bootstrapping was performed using the Statistical Analysis Software (SAS, version 9.2).

## Results

**Figure 1:**
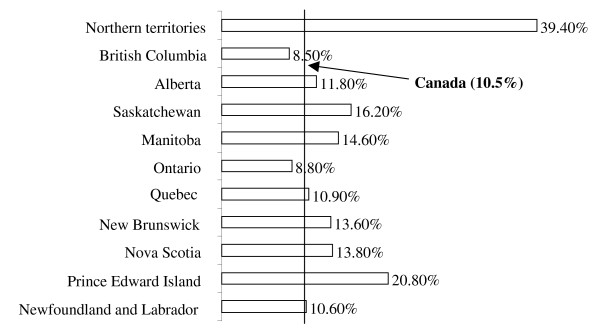
**Distribution of smoking during pregnancy across the Canadian provinces and territories (2005/06)**.

The sample size for the population analyzed in this study was 6,421 weighted to represent 76,508 Canadian women. Table 1 presents the estimated population and distribution of smoking practices before, during and after pregnancy. The proportion of women who smoked before, pregnancy was 22.0%, while it was 16.5% after pregnancy. The prevalence of smoking during pregnancy, however, was 10.5% (6.9% daily smokers and 3.6% occasional smokers). Women who smoked during pregnancy consumed on average a total of 7 cigarettes a day (95% CI: 6.5-7.4; SD = 5.7), whereby daily users smoked on average 9 cigarettes (95% CI: 8.4-9.5; SD = 5.9) and occasional users smoked on average 3 cigarettes (95% CI: 2.9-3.5; SD = 2.5). Most regions in Canada displayed relatively similar smoking rates during pregnancy except for the Northern Territories (39.4%) and Prince Edward Island (20.8%) (Figure 1). The prevalence of smoking, on the other hand, was least prevalent in British Columbia (8.5%) and Ontario (8.8%).

**Table 1:** Distribution of smoking before, during and after pregnancy among Canadian mothers (2005/06).

	N*	% (95% CI)†
Smoking before pregnancy	16,823	22.0 (21.1-23.0)
Smoking after pregnancy	12,634	16.5 (15.7-17.4)
Smoking during pregnancy	8,015	10.5 (9.8-11.2)
Smoking during pregnancy frequency		
Daily	5,253	6.9 (6.3-7.5)
Occasionally	2,762	3.6 (3.2-4.1)
Never	68,319	89.5 (88.8-90.2)

* Sample size is estimated using population weights

† 95% CI were calculated using bootstrapping technique

Unadjusted and adjusted associations between smoking during pregnancy and potential factors are illustrated in Table 2. All variables significant at the bivariate level were considered for a logistic regression model. Out of the 18 significant variables at the bivariate level, 10 remained significant in the final model. Income, place of residence and years of education, that are considered to be indicators of socio-economic status, were negatively associated with smoking during pregnancy. Mothers who had the lowest household income (<$30,000 and $30,000 to less than $60,000) were more likely to smoke during pregnancy (OR: 1.73, 95% CI: 1.08-2.78 and OR: 1.86, 95% CI: 1.21-2.85, respectively) than women with higher income. Similarly, women who lived in rural and semi-urban areas had higher odds of smoking in their pregnancy. In addition to socio-economic status, marital status was found be significantly associated with smoking during pregnancy, whereby single mothers were more prone to smoking. Although old mothers were more likely to be smokers, mothers who had their first pregnancy at a young age were also at an increased risk of smoking during pregnancy. Moreover, non-immigrant mothers and passive smokers were almost 5 times more likely to smoke during their pregnancy as compared to their counterparts (95% CI: 3.32-8.51 and 95% CI: 4.28-6.58, respectively). Finally, the failure to attend prenatal classes and experiencing stressful events were found to be associated with smoking during pregnancy. The odds ratio of not attending prenatal classes was 1.43 (95% CI: 1.10-1.86), while it was 1.15 for experiencing stressful events (95% CI: 1.08-1.23).

**Table 2:** Unadjusted and adjusted associations between smoking during pregnancy and potential predictors.

	Sample size	Smoking during pregnancy	Unadjusted odds ratio	Adjusted odds ratio
	N*	N*(%)	OR (95% CI)†	OR (95% CI) †
Household income				
< $30,000	1,031	230 (22.3)	**10.12 (6.77-15.13)**	**1.73 (1.08-2.78)**
$30,000 to less than $60,000	1,853	242 (13.1)	**5.31 (3.54-7.95)**	**1.86 (1.21-2.85)**
$60,000 to less than $100,000	1,940	111 (5.7)	**2.14 (1.39-3.30)**	1.09 (0.71-1.69)
≥ $100,000	1,212	33 (2.7)	1	1
Place of residence				
Rural area	1,100	150 (13.6)	**2.23 (1.78-2.80)**	**1.40 (1.04-1.87)**
Urban, population ≤ 499,999	2,283	310 (13.6)	**2.22 (1.81-2.72)**	**1.42 (1.10-1.82)**
Urban, population ≥ 500,000	2,791	185 (6.6)	1	1
Immigrant				
No	4,980	641 (12.9)	**7.26 (4.79-11.00)**	**5.32 (3.32-8.51)**
Yes	1,407	28 (2.0)	1	1
Work during pregnancy				
No	1,364	195 (14.3)	**1.60 (1.34-1.91)**	0.93 (0.72-1.20)
Yes	5,028	475 (9.4)	1	1
Marital status				
No partner	535	168 (31.4)	**4.88 (3.97-6.01)**	**1.83 (1.33-2.53)**
Have a partner	5,860	501 (8.5)	1	1
Moms perceived health				
Excellent/very good	4,645	396 (8.5)	1	1
Good	1,419	208 (14.7)	**1.84 (1.53-2.21)**	1.17 (0.92-1.49)
Poor/Fair	341	69 (20.2)	**2.70 (2.03-3.60)**	1.01 (0.67-1.51)
Alcohol drinking during pregnancy				
No	5,713	576 (10.1)	1	1
Yes	669	89 (13.3)	**1.36 (1.06-1.74)**	1.28 (0.93-1.77)
Health problems during pregnancy				
No	4,837	485 (10.0)	1	1
Yes	1,563	186 (11.9)	**1.21 (1.01-1.46)**	1.19 (0.95-1.50)
Reaction when discovered pregnancy				
Very happy/happy	5,940	576 (9.7)	1	1
Indifferent	265	58 (21.9)	**2.63 (1.94-3.56)**	1.09 (0.71-1.69)
Very unhappy/Unhappy	180	36 (20.0)	2.35 (1.60-3.46)	0.93 (0.54-1.61)
Attended prenatal classes				
No	4,306	528 (12.3)	**1.89 (1.56-2.28)**	**1.43 (1.10-1.86)**
Yes	2,097	144 (6.9)	1	1
Support during pregnancy				
None/Little of time	328	50 (15.2)	**1.59 (1.15-2.21)**	1.25 (0.82-1.91)
Some of the time	502	52 (10.4)	1.03 (0.76-1.39)	0.97 (0.65-1.46)
Most/All of time	5,555	567 (10.2)	1	1
Passive smoking during pregnancy				
No	4,903	206 (4.2)	1	1
Yes	1,499	466 (31.1)	**10.28 (8.64-12.23)**	**5.31 (4.28-6.58)**
Province‡				
Eastern-Atlantic	379	51 (13.5)	1	1
Eastern-Central	4,018	385 (9.6)	**0.69 (0.57-0.83)**	1.27 (0.85-1.89)
Western-Prairies	1,220	159 (13.0)	0.97 (0.78-1.21)	1.15 (0.74-1.76)
Western-British Columbia	755	64 (8.5)	0.60 (0.44-0.82)	1.24 (0.76-2.02)
Northern territories	33	13 (39.4)	**4.09 (3.32-5.05)**	2.29 (0.73-7.14)
		**Unadjusted Mean difference^§ ^(95% CI)†**		
Years of education	6,349	**-2.57 (-2.77--2.36)**		0.84 (0.80-0.88)
Age at first pregnancy	6,332	**-4.89 (-5.31--4.47)**		0.93 (0.90-0.96)
Number of past pregnancies	6,397	**0.62 (0.46-0.77)**		1.08 (0.99-1.18)
Mother's age at selected birth	6,368	**-2.88 (-3.36--2.39)**		**1.04 (1.01-1.07)**
Weight gained during pregnancy	6,332	0.50 (-0.20-1.21)		--
Number of stressful events	6,353	**1.25 (1.07-1.42)**		**1.15 (1.08-1.23)**
Number of prenatal visits	6,138	0.20 (-0.23-0.64)		--

* Sample size is estimated using normalized weights

† 95% CI were calculated using bootstrapping technique

‡ Eastern Atlantic: Newfoundland & Labrador, Nova Scotia, Prince Edward Island & New Brunswick; Eastern Central: Quebec & Ontario; Western Prairies: Manitoba, Saskatchewan, & Alberta; Western British Columbia: British Columbia; and Northern Territories: Yukon Territory, Nunavut & Northwest Territories.

^§ ^Represents the difference between the mean of smokers and non-smokers (Mean_smokers _- Mean_non-smokers)_.

## Discussion

The present study aimed to assess the prevalence and characteristics of smoking during pregnancy among Canadian women. Results showed that the prevalence of smoking at any time in the third trimester was 10.5%, and that the average daily consumption of cigarettes was around 7 (95% CI: 6.5-7.4; SD = 5.7). Regression analysis revealed that mothers who smoked during pregnancy were at greater odds for being of low socio-economic status, non-immigrant, single and exposed to passive smoking during pregnancy. Not attending prenatal classes and experiencing stressful events before and during pregnancy also increased the mothers' odds of smoking during pregnancy. While the age of the mother's first pregnancy was negatively associated with smoking during pregnancy, the mother's current age was positively associated with it.

The prevalence of smoking during pregnancy is comparable with other modern industrialized nations. A recent American study reported that the smoking rate is 10% [37], while another study from Germany assessed the rate as 13% [38]. The present study's smoking rate, however, is lower than the previous two national Canadian studies [13,22]. Connor & McIntyre (1999) determined the prevalence rate of smoking during pregnancy in 1994 as 23.7% and Millar & Hill (2004) evaluated it as 17% in 2000/01. The provincial prevalence rates of the present study are also lower than those reported by Millar & Hill (2004). In Newfoundland and Labrador, for example, the rate was previously described as 26% while it was revealed to be 10.6% in the present study. Similarly, the rate dropped from 17% to 13.8% in Nova Scotia, from 21% to 10.9% in Quebec, from 14% to 8.8% in Ontario, and from 18% to 16.2% in Saskatchewan. The smoking rates in Alberta and British Columbia were also reported to be lower than 2000/01 rates. These differences in rates can either be attributed to variations in study designs, sample selection and variable definitions or to an actual decline in the rate of smoking during pregnancy in Canada. The decline that was observed in this study is paralleled by a similar decline in the overall smoking rates in the Canadian adult population. Based on data from the mid-1960s to the 1994/1995, the overall rate of smoking in Canada has dropped from 45% to 31% [39]. In another study, the rate of smoking in 2003 was reported to be 21% [40]. The reason for this decline might be due to the collective effects of legislations on smoking bans and increased awareness on the harmful consequences of smoking.

An alarming result of the study is the high prevalence (39.4%) of smoking among women in the Northern territories. A previous study of 162 women seeking prenatal care at 10 communities in the Inuvik Zone, North West Territories, who gave birth between 1987 and 1990, noted that 64% of the women smoked during pregnancy [41]. These high rates may be attributed to the high use of tobacco among the aboriginal population. Tobacco, among some of the aboriginals, is commonly used in ceremonies, prayers or other cultural rituals [42]. Poverty and unemployment in these communities can be another possible reason for these high rates [41].

Based on the multivariate analysis, smoking during pregnancy was associated with low socio-economic status. This finding is consistent with other studies [43,44]. Millar & Hill (2004) reported a dose response relationship between smoking during pregnancy and household income. The indictors of low income status were also associated with higher smoking prevalence in Ontario [23], Saskatchewan [20] and Manitoba [15].

In the present study, age of the mother's first pregnancy was negatively associated with smoking during pregnancy whereas her current age was positively associated with smoking during pregnancy. A Canadian study of 1134 women done by Johnson et al. (2004) found that smokers during pregnancy were 2.4 times (95% CI: 1.5-3.8) more likely to be under 25 years of age [14]. Connor and McIntyre (1999) also found that women aged 15-24 years tended to relapse to smoking during their pregnancies [22].

According to the study findings, Canadian-born women were more likely to smoke during pregnancy than immigrants. This finding has also been noted in previous studies [23]. Johnson et al. (2004) noted that Canadian-born women were at 4.2 (95% CI: 2.6-7.0) increased odds of smoking than those born outside of Canada [14]. Based on the 2000/01 national study, 22% of non-immigrant mothers were smoking during pregnancy as compared to 2% of immigrant mothers. Moreover, Canadian studies tend to mirror the results of the present study regarding increased risk of smoking during pregnancy among single women [20]. A Manitoban study found that the odds ratio for smoking during pregnancy among single non-Aboriginals and Aboriginals to be 3.23 (95% CI: 1.44-7.23). Similarly, the national percentage of unmarried smokers (34%) was more than twice than married smokers (14%) [13]. Perhaps contributing to higher smoking rates in single women is the present study's assertion that stressful events before and during pregnancy help trigger the up-take of smoking.

Perhaps unsurprisingly, women who were exposed to passive smoking during their pregnancy were more likely to smoke. This finding was supported by Paterson et al. (2003) which found that pregnant smokers were more likely to have others in the household who also smoked as compared to quitters of smoking [21]. A study in Ottawa also found that among pregnant smoking women, 76% had a smoking partner and 38% were exposed to smoking in the workplace, whereby only 15% of non-smokers had a partner who smoked, and only 13% reported workplace exposure [28].

Attending prenatal classes was found to be significantly associated with smoking during pregnancy in this study. Prenatal classes is undoubtedly an important educational opportunity to provide information to would-be mothers on the effects of smoking during pregnancy and cessation programs [25]. An older Canadian study stated that among primiparous women, prenatal classes were attended by 61.6% of smokers compared with 85.6% of non-smokers [24]. Likewise, a study of Canadian nulliparous women stated that those who attended prenatal classes were less likely to smoke during pregnancy [25].

This study makes noteworthy contributions to knowledge about factors related to smoking during pregnancy. The ability to adjust for many individual level factors that may affect smoking during pregnancy and to quantify smoking consumption, yields to more accurate findings. Although self-reporting in questionnaires are subject to recall bias, it is kept minimal in this study due to the short time lapse from pregnancy to data collection.

There are, however, limitations to this study. The response rate in the present study was 75.2%. The main reason for non-response was the inability to establish contact with the mothers who were initially selected from the Canadian Census of Population. However, the population weights created by Statistics Canada and used in the analysis accounted for this non-response. Moreover, the main outcome variable, having ever smoked in the last 3 months of pregnancy, may not fully capture the true smoking habits of these women. Haman and Chalmers (2005) found that both Aboriginal and non Aboriginal women tended to stop smoking as the pregnancy progressed [15]. Another Canadian study found that, smoking rates in the first, second and third trimesters, decreased respectively from 9.6% to 8.7% and 8.1% [14]. A study done in Ottawa found that, by the end of the first trimester, 28.5% of women smoked. This number was reduced to 26% by the time of delivery [24]. Based on the latter studies, the definition employed in the present survey may fail to capture all smoking mothers during pregnancy. Therefore, the study rates are believed to be lower than the actual rates of ever smoking during pregnancy. The study also relies on self-reports of several sensitive variables such as smoking and drinking during pregnancy. Consequently, these women would be inclined to socially desirable reporting.

## Conclusion

Although the rate of smoking during pregnancy is lower than the last Canadian study, it is still prevalent among 10% of the Canadian mother population. The findings of the study may be useful to design prevention programs that target specific themes and/or populations. Understanding the attitudes, practices and opinions of pregnant women in the Northern Territories is highly warranted. By doing so, more effective interventions can be tailored to meet their needs. Moreover, more efforts to increase women's participation in prenatal classes is recommended. Prenatal classes are a fertile ground for raising awareness and promoting smoking cessation programs. Finally, since stress and smoking have been found to be associated, future studies can investigate the effect of stress reduction on smoking rates during pregnancy.

## Abbreviations

CI: Confidence interval; MES: Maternity Experience Survey; OR: Odds Ratio; SAS: Statistical Analysis Software; SD: Standard deviation; SPSS: Statistical Package for Social Sciences.

## Competing interests

The authors declare that they have no competing interests.

## Authors' contributions

BAS performed the analysis and contributed in writing the manuscript. MS assisted in the literature review and write up of the manuscript. GH assisted in the literature review and write up of the manuscript. HT generated the idea of the research and supervised the analysis and write up of the manuscript. All authors read and approved the final manuscript.

## Pre-publication history

The pre-publication history for this paper can be accessed here:

http://www.biomedcentral.com/1471-2393/10/24/prepub
